# Associations between Flavonoid Intakes and Gut Microbiota in a Group of Adults with Cystic Fibrosis

**DOI:** 10.3390/nu10091264

**Published:** 2018-09-07

**Authors:** Li Li, Shawn Somerset

**Affiliations:** 1 School of Medicine, Menzies Health Institute Queensland, Griffith University, 68 University Drive, Meadowbrook, QLD 4131, Australia; li.li14@griffithuni.edu.au; 2 Faculty of Health, University of Canberra, University Drive, Bruce, ACT 2617, Australia

**Keywords:** flavonoids, cystic fibrosis, gut microbiota, inflammation

## Abstract

Dietary flavonoid intakes can influence gut microbiota (GM), which in turn can affect immune function and host metabolism, both vital considerations in cystic fibrosis (CF) management. In CF, GM may be altered and link to CF respiratory events. This study explored the relationship between flavonoid intakes and GM in free-living adults with CF. Associations between the overall GM variations (unweighted and weighted UniFrac distances between pyrosequencing results of bacterial 16-ss rDNA from frozen faecal samples of sixteen CF adults) and standardised dietary flavonoid intakes (a validated flavonoid-specific food frequency questionnaire) were analysed using adonis tests. Flavonoid intakes that were significant at a false discovery rate (FDR) < 0.3 were subjected to Spearman correlation tests with standardised bacterial relative abundances (FDR < 0.3). Gallocatechin intakes (*p* = 0.047, *q* = 0.285) were associated with unweighted UniFrac distances. Intakes of apigenin (*p* = 0.028, *q* = 0.227) and kaempferol (*p* = 0.029, *q* = 0.227), and % flavonoid intake as flavones (*p* = 0.013, *q* = 0.227) and flavonols (*p* = 0.016, *q* = 0.227) (both excluding contribution of tea) were associated with weighted UniFrac distances. Among these, gallocatechin correlated with the genus *Actinomyces* and family *Actinomycetaceae* (*Actinobacteria*). Gallocatechin correlated negatively with class *Coriobacteriia* (*Actinobacteria*). Intakes of some flavonoids may be associated with GM variations with potential consequences for metabolism, immune function, and inflammation, which are important in CF lung disease and co-morbidity management.

## 1. Introduction

Survival of people with cystic fibrosis (CF) has improved remarkably with advanced treatment over the past few decades [1]. Concomitantly, a rising trend has been observed in the prevalence and risk of CF-related comorbidities such as CF-related diabetes (CFRD) [2] and gastrointestinal malignancy including colorectal cancer [1,3]. Pre-emptive strategies to address these risks therefore need to be incorporated into current therapies. Gut microbiota and their metabolites can influence host metabolism and immune function [4,5,6,7], which are heavily implicated in CF progression and comorbidity management [8]. For example, colorectal cancer, as a comorbidity of CF, seems to be associated with a distorted gut microbiota in the general population [9]. In CF, gut microbiota also appears altered [10,11,12]. Such alteration may even be linked to pulmonary exacerbations and *Pseudomonas aeruginosa* colonisation [13]. The potential involvement of gut microbiota in CF disease progression and management is thus implicated.

Limited preliminary studies have shown that oral probiotics may help reduce pulmonary exacerbation frequencies and associated hospital admissions [14,15], and gut inflammation and discomfort [10,16] in children and adults with CF [17]. It is thus plausible that modulation of gut microbiota in CF may enhance current treatment. However, there seems to be a paucity of such data, potentially due to the lack of more prominent clinical improvement in those probiotic trials when compared with recent CFTR modulator therapies [18,19]. Participation in gut microbiota modulation studies may also be considered as unnecessary given the existing high treatment burden [20].

The effect of dietary modulation of gut microbiota has to date focussed on macronutrients and non-digestible carbohydrates [21]. Evidence is emerging that non-nutrient dietary constituents such as flavonoids can also influence gut microbiota composition [22,23,24]. Population, clinical, and mechanistic studies have also highlighted the association of flavonoid intake with various inflammation-associated chronic conditions such as diabetes and certain cancers including colorectal cancer, at least partially mediated by gut microbial metabolism of various flavonoids [25,26]. CF is characterised by inflammation, metabolic abnormality such as CFRD and increased risk of malignancy [1,27]. Flavonoids may thus contribute to the management of CF and comorbidities potentially via modulation by gut microbiota. Investigation on relationships between these dietary flavonoids and gut microbiota in CF has not been reported previously. This study thus explored associations between intakes of dietary flavonoid intakes and gut microbiota composition in a group of free-living adults with CF. The results are considered candidate flavonoids, whose therapeutic potential in CF management requires further examination.

## 2. Materials and Methods 

Eighteen free-living adults with stable CF and signed informed consent were recruited with the help of CF community support organisations in Brisbane and Sydney, Australia. They were clinically stable, which was was defined as having no pulmonary exacerbations, no overnight hospital admission, and no body weight change > 3% of their body from 8 weeks prior to commencement of the study weight until commencement of the study [28,29]. Prior to and during the study, participants were free from cardiac disease, not pregnant, with a bowel frequency between once every two days and three times per day [24]. They were not taking tricyclic antidepressants, narcotics, antacids, anti-diarrhoea medications within four weeks prior to enrolment, and none had been smoking or on total parental nutrition. Those using antibiotics, laxatives, proton pump inhibitors, H_2_ receptor antagonists, or anticholinergic medications were not excluded, as these are common CF therapeutics [30]. The study was approved by the University Human Research Ethics Committee (Ref No: PBH/39/11/HREC). 

Methods of subject recruitment, dietary and clinical information collection, and microbiota analysis have been reported previously [31]. Dietary data other than flavonoids were reported by participants via food diaries on three consecutive days including one weekend day just before faecal sample collection. A validated flavonoid-specific food frequency questionnaire (FFQ) [32] (supplementary notes) was telephone-administered to estimate participants’ flavonoid intakes over the year prior to study. A faecal sample from each participant was collected and transported in insulated bags with pre-frozen ice packs and stored at −20 °C before transportation to storage at −80 °C. The storage at −20 °C lasted between a couple of days to around ten days. Participants also self-reported demographic and clinical data as summarised in Table S1. The results on flavonoids were analysed and reported separately from other dietary variables because they were collected using different methods. 

These faecal samples were then transported on dry ice for DNA extraction and sequencing at the Australian Genome Research Facility (AGRF Ltd, Brisbane, Australia). DNA extracted from approximately 200 mg of each frozen faecal sample was sequenced using primers (Table S2) targeting the V1-V3 hypervariable regions of the bacterial 16 small subunit ribosomal DNA using 454 pyrosequencing. Sequences were analysed and taxonomy assigned using the Quantitative Insights Into Microbial Ecology (QIIME) software package version 1.8 (http://qiime.org/) following default procedures and settings [33]. The sequencing results were, however, demultiplexed and assigned taxonomic identities using open-reference OTU (operational taxonomic unit) picking against the Greengenes OTUs database dated May 2013. Reverse primers and chimeric sequences were removed. Taxonomy of the sequencing results with the respective absolute and relative abundances were summarised at the genus, family, order, class, and phylum levels for each sample. Pairwise weighted and unweighted UniFrac distances [34] between samples were calculated after single even-depth rarefaction based on the minimal number of sequences generated among all samples (depth = 2466). Alpha diversity including chao1 and Shannon Index of each sample was also calculated and plotted. 

One participant withdrew from the study due to another unspecified commitment and one faecal sample was eliminated during quality control for sequencing, leaving sixteen samples for downstream analyses. 

Flavonoid intakes estimated by the flavonoid-specific FFQ [32] are summarised in Table S3. Percentage contributions of flavonoid sub-classes including flavonols, flavones, flavan-3-ols, flavanones, and anthocyanidins to flavonoid intakes excluding tea were also determined.

Estimates of flavonoid intakes were standardised (*z*-scores) across samples. The overall association between gut microbiota variations (as represented by unweighted and weighted UniFrac distances [34]) among participants and flavonoid intakes were tested using adonis tests [35] adjusted for multiple testing at a false discovery rate (FDR) < 0.3 [36]. Spearman correlation tests were used to examine the correlations between flavonoids significant at FDR < 0.3 were subjected to the Spearman correlation test with standardised relative abundances of taxa at the genus, family, order, class, and phylum levels adjusted for multiple testing at an FDR < 0.3. Statistical analyses were carried out using R (version 3.1.1, R Foundation for Statistical Computing, Vienna, Austria). Adjustment for multiple testing at an FDR <0.1 was attempted with nil significant results. A less stringent FDR threshold was applied to not miss any candidate variable [24] in this exploratory study. This approach has been adopted by other studies investigating candidate factors associated with gut microbiota [24,37].

The association between gut microbiota and intakes of macronutrients (including energy, dietary fibre, and resistant starch) and micronutrients followed the same process. The association between gut microbiota and use of medications such as antibiotics, laxatives, and proton pump inhibitors, and other clinical characteristics such as age, gender, BMI, pulmonary function, and use of probiotics was tested using the same approach. The relationship between gut microbiota and micronutrients was previously reported [31]. Other results will be reported separately. 

The association between alpha diversity indices at various taxonomic levels and flavonoid intakes was attempted using ANOVA (online software Calypso [38]), with nil significant results and hence not analysed further.

## 3. Results

Details of participants’ characteristics are summarised in supplementary Table S1. The predominant phylum in the gut microbiota (Table S4) was *Firmicutes* (86.4%), followed by *Bacteroidetes* (5.6%), *Actinobacteria* (5.1%), unassigned (1.7%), and *Proteobacteria* (1.3%), despite inter-individual differences (Figure S1). Alpha diversity as represented by chao1 and Shannon indices can also be found in Figure S1.

Associations between flavonoid intakes and overall gut microbiota variations among the participants at an FDR < 0.3 are present in Table 1 and Table 2. Among the flavonoids (Table S3) tested, only gallocatechin intake was associated with variations in the presence/absence of bacterial taxa as represented by unweighted UniFrac distances [34]. Intakes of apigenin and kaempferol, and contribution of flavones and flavonols to flavonoid intakes (excluding tea contribution) were associated variations in the presence/absence of bacterial taxa as represented by weighted UniFrac distances [34]. Intakes of flavonoids identified based on weighted UniFrac distances (Table 2) showed stronger associations (larger *R*^2^ values) with gut microbiota variations than those identified using unweighted UniFrac distances (Table 1). 

At an FDR < 0.3, weighted UniFrac distances were also correlated with gender and use of inhaled antibiotics. These results will be reported and discussed in a separate analysis, together with other variables. Due to the limited sample size, it was not feasible to adjust the associations with flavonoids using these other variables.

**Figure 1:**
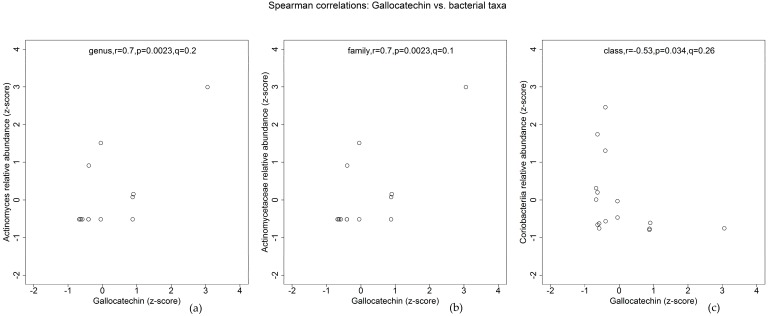
Spearman’s correlations between specific gut bacteria taxa and gallocatechin intake in a group of free-living adults with cystic fibrosis (CF). Positive correlations between gallocatechin intake and *Actinomyces* (**a**) and *Actinomycetaceae* (**b**), negative correlation between gallocatechin intake and *Coriobacteriia* (**c**); *r* = Spearman’s rho, *p* = raw *p* values, *q* = *p* values adjusted for multiple testing at an FDR < 0.3.

Among the aforementioned flavonoids that were associated with overall gut microbiota variations, only gallocatechin, a major black tea flavonoid, was found to correlate with specific bacterial taxa that belong to the phylum *Actinobacteria* (Figure 1 and Figure S2). Gallocatechin intakes correlated positively with *Actinomyces* and *Actinomycetaceae* (*Actinobacteria*), but negatively with the class *Coriobacteriia* (*Actinobacteria*). The latter was composed of predominantly *Coriobacteriaceae* members, correlation of which with gallocatechin intake was similar to *Coriobacteriia* but became insignificant at an FDR of 0.3 (*r* = −0.53, *p* = 0.034, *q* = 0.31).

There did not seem to be any correlations between use of oral antibiotic therapy and the relative abundances of *Actinomyces* (*r* = 0.15, *p* = 0.57), *Actinomycetaceae* (*r* = 0.15, *p* = 0.57) or *Coriobacteriia* (*r* = −0.031, *p* = 0.91). Thus, the use of oral antibiotics did not seem to influence the associations between gallocatechin and these gut bacterial taxa. 

## 4. Discussion

The present study found associations between intakes of specific flavonoids and gut microbiota in a small group of free-living adults with CF. It should be noted that the current study was part of a broader study on the relationship between various dietary constituents and gut microbiota in CF. Consequently, the results of the study should also be viewed in a broader dietary and clinical context specific to CF. 

In this analysis, flavonoids associated with relative abundances (weighted UniFrac distances) of gut bacteria taxa (Table 2) differed from those associated with the presence/absence (unweighted UniFrac distances) of this community (Table 1). Moreover, the associations observed based on weighted UniFrac distances appeared to be stronger than those observed based on unweighted UniFrac distances. This indicates that different flavonoids may associate with variation in either relative abundances or presence/absence of gut bacterial taxa in CF, and the associations with relative abundances may be stronger. The lack of associations between alpha diversity indices and flavonoid intakes implied that the intakes were not associated with community member abundance or evenness in each sample.

The significant associations between intakes of specific flavonoids and gut microbiota variations are notable. Flavonoid intakes data were collected using a flavonoid-specific FFQ [32] intended to assess usual longitudinal intakes. In contrast, participant cross-sectional gut microbiota profiles may fluctuate according to various host and environmental factors including diet [39,40,41]. Thus, the significant associations between intakes of specific flavonoids and gut microbiota variations observed imply their potential long-term associations and possibly long-term influence of such flavonoids on gut microbiota in CF. Such a relationship is partially supported by the long-term stability [42] and the relatively stable overall inter-individual variations in gut microbiota in response to short-term identical dietary changes [24] in the general population. 

In particular, the phylum *Actinobacteria* seems to be more stable than *Firmicutes* [42], the predominant phylum in the present study. Since gallocatechin intakes correlated positively with *Actinomycetaceae* and its lower rank genus *Actinomcyes* (*Actinobacteria*), but negatively with *Coriobacteriaceae* (*Actinobacteria*) (Figure 1), it is speculated that gallocatechin intakes, of which the predominant dietary source was black tea (Figure S3), may be linked to long-term status of these *Actinobacteria* members in the gut microbiota in adults with CF. The associations of other black tea flavonoids such as thearubigins and theaflavin and its derivatives with overall gut microbiota variations approached significance (Table 1 and Table 2). Furthermore, other tea flavonoid intakes (e.g., epicatechin and derivatives), of which the main dietary sources are not limited to black tea (Figure S3), were not associated with UniFrac distances. These observations suggest that black tea and its major flavonoids may correlate positively with the relative abundance of *Actinomcyes* and negatively with that of *Coriobacteriia,* which in this study comprised only taxa from the family *Coriobacteriaceae*. This is interesting in view of previous in vitro studies using human [43,44] and/or rat faecal bacteria [44]. Specific *Coriobacteriaceae* strains were found to metabolise epicatechin, catechin [43] and gallocatechin [44]. However, the presence of *Actinomcyes* spp. was unspecified in one study [43] and the other focused only on four strains of *Coriobacteriaceae* in the absence of *Actinomcyes* spp. [44]. 

The role of gut *Actinomyces* and *Coriobacteriaceae* in CF remains undefined, probably because of their lower relative abundances in human gut microbiota studies [45]. However, accumulating evidence suggests a role in macronutrient metabolism, energy homeostasis, infection, and acute immune response in humans [45,46,47,48]. Moreover, *Actinomyces*, together with other anaerobes has been shown to be enriched in a large proportion of CF sputum samples, particularly concurrent with *Pseudomonas aeruginosa* colonisation and possibly related to different antibiotic regimens [49]. Whether a similar situation occurs in the gut due to antibiotic usage is yet to be confirmed, but the potential link between the gut and respiratory microbiomes in CF [50] indicates a need to evaluate the correlation of *Actinomcyes* with black tea flavonoids. The role of *Coriobacteriaceae* in CF remains unclear, but faecal *Coriobacteriaceae* levels are elevated in those with colorectal cancer compared with healthy controls [51]. Assuming *Actinomcyes* and *Coriobacteriaceae* influence the status of CF lung disease and colorectal cancer (CRC), and black tea and its flavonoids can indeed modify the gut microbiota in CF, modified nutritional therapies/recommendations incorporating this could have therapeutic potential to complement current management and/or reduce risk of lung disease and CRC in CF. The results presented here stress the need to further investigate the role of black tea consumption in CF nutrition therapy, in view of black tea being a major dietary flavonoid source in a western diet [52].

Despite the absence of significant correlations with specific bacterial taxa, intakes of apigenin and kaempferol were associated with overall gut microbiome variations based on weighted UniFrac distances (Table 2). The same was observed for contribution of flavones (including apigenin and luteolin) and flavonols (including quercetin, isorhamnetin, kaempferol and myricetin) to total flavonoid intakes, excluding contribution of tea flavonoids. Such associations are supported by in vitro studies, in animal [23] and human models [53]. Several mechanisms have been proposed for the influence of polyphenols including flavonoids such as flavones and flavonols on the gut microbiota [54]. These include disrupting bacterial cell-wall/membrane components to impede growth, modulate production of virulence factors, interfering quorum sensing, and supressing bacterial nucleic acid biosynthesis [55]. The potential implications of associations between these flavonoids and gut microbiota in CF management await further investigation. However, flavone intakes may be associated with *Blautia* that is involved in up-regulation of regulatory T cells [53]. The same study also demonstrated negative associations between flavonol intakes and *Bifidibacterium* (phylum *Actinobacteria*). Interestingly, an in vitro study observed that flavonols may increase synthesis of the anti-inflammatory nitric oxide in one *Bifidibacterium* species upon stimulation by lipopolysaccharide [55]. Lipopolysaccharide, contained in the cell wall of gram-negative bacteria such as *P*. *aeruginosa*, has been shown to contribute to chronic inflammation in the general population [56], and respiratory inflammation [57,58] and pancreatic exocrine and endocrine dysfunctions mediated by inflammatory pathways involving nuclear factor-κB [59] in CF. Recurrent inflammation and chronic respiratory colonisation by *P*. *aeruginosa* are common in CF [57]. Thus, associations between flavonoid intakes and gut microbiota variations in CF may have implications in management of inflammation-mediated CF co-morbidities. 

The generalisability of our observations to the wider CF population is limited by the small sample size, the cross-sectional setting, and the specific methods and conditions in handling and processing faecal samples and sequencing. Inclusion of gut mucosal microbiota samples may also produce different sequencing results [60,61,62,63,64,65]. All *q* values were >0.1, potentially reflecting the lack of power due to the small sample size. Despite such limitations, this exploratory study was carried out to screen for potential dietary components including candidate flavonoids that may modulate the gut microbiota and influence clinical outcomes of CF. Further studies with a larger sample size and gender-matched healthy controls are warranted to determine whether the flavonoid candidates identified in the present study can modify the gut microbiota in CF.

Although use of FFQ to estimate flavonoid intakes may not be quantitatively accurate, for non-parametric testings of associations and correlations, the ranking of flavonoid intakes rather than absolute intakes was more relevant. Greater than 85% of participants were placed to the same or adjacent quartile group using the FFQ for total flavonoids, and greater than 73% for the majority of the individual flavonoids included and all flavonoid subgroups except one, when compared with data collected by seven-day measured food records [32]. Therefore, it was considered appropriate to use this flavonoid-specific FFQ to assess flavonoid intakes for the present study.

The FFQ was used to estimate the habitual intakes of flavonoids. Long-term associations between dietary intakes and clusters of gut microbiota called enterotypes in the general population have been reported [24]. Thus, clustering the gut microbiota profiles in the current study into enterotypes was attempted according to Arumugam et al. [66] and Koren et al. [67]. Stable enterotype clustering was, however, not observed in the present study, probably due to the small sample size, as shown by low Silhouette widths (mostly <0.3, recommendation >0.75 [67]) and Calinksi-Harabasz scores indicating >10 number of clusters. The relationship between flavonoid intakes and gut enterotypes in CF thus needs to be investigated in larger CF cohorts.

These observed associations between certain flavonoids and gut microbiota in the present study warrant further evaluation in larger CF cohorts, preferably in longitudinal settings, on their potential implications in management of CF lung disease and its ageing- and inflammation-associated co-morbidities such as CFRD and colorectal cancer. Prevalence of these co-morbidities has increased with the much improved survival in CF [1]. Interestingly, the phylum *Actinobacteria* has been found previously to be more stable than the predominant *Firmicutes* phylum in humans [42]. Also, the relationship between intakes of other flavonoids (apigenin, kaempferol, % flavone, and % flavonol (both excluding tea contribution)), and the gut microbiota in CF needs further investigation, particularly if they also correlate with specific gut bacterial species, considering their aforementioned potential involvement in inflammation and immune regulation. The difficulties in determining causality of dietary components such as flavonoids as modulators of human gut microbiota and hence downstream physiological effects cannot be underestimated [68]. However, faecal transplants from human donors to animal models have demonstrated its usefulness in confirming the role of dietary modulation of gut microbiota in some disease models. Other aspects to be considered in further investigation on effects of dietary modulation of gut microbiota in CF include the influence of other gut microbiota modulating factors such as antibiotics [69] and gender [40] on the effect of dietary modulation of gut microbiota by flavonoids, differences in metabolism between strains of the same gut bacterial species [68], the potential adaptation of phenotypes of particular bacterial species to the CF intestinal environment [70], and variable individual responses (including timeframe and the extent of changes) to dietary modulation of gut microbiota partially due to different individual baseline diets [21]. There seems to be associations between gut microbiota variation and some micronutrients in CF [31]. If both flavonoids and certain micronutrients can modify gut microbiota in CF, it remains to be determined whether the effect is due to individual dietary component or a combination of two or more dietary constituents. The effect size (*R*^2^) of the adonis tests (FDR < 0.3) (Table 1 and Table 2) were similar between the flavonoids and the micronutrients tested [31]. Since micronutrient intake data were recorded in food diaries for the three days prior to the collection of the faecal samples, overall variation in the gut microbiota associated with flavonoid intake recorded in a flavonoid-specific FFQ seemed to be similar to that associated with micronutrient intake recorded in food diaries prior to faecal sample collection.

Diet is among the most modifiable factors that can shape the gut microbiota [24]. Further understanding of the gut microbiota modulatory effect of dietary constituents such as flavonoids may help develop more holistic nutritional therapies targeting multiple CF-related conditions to further improve the quality of life in CF.

## Figures and Tables

**Table 1:** Associations between flavonoid intakes ^1^ and gut microbiome based on unweighted UniFrac distances.

Variable	*p* Value ^2^	*R*^2^ ^2^	*q* Value ^3^
Gallocatechin	0.047	0.078	0.285
Theaflavin digallate	0.051	0.078	0.285
Thearubigins	0.051	0.078	0.285
Theaflavin	0.052	0.078	0.285
Theaflavin-3′-gallate	0.052	0.078	0.285
Theaflavin-3-gallate	0.055	0.078	0.285
% Anthocyanidins (excluding tea)	0.056	0.077	0.285
Petunidin	0.087	0.076	0.387
Peonidin	0.101	0.075	0.387
Malvidin	0.108	0.076	0.387
Eriodictyol	0.157	0.075	0.514
Wine flavonoids	0.186	0.074	0.514
Cocoa flavonoids	0.186	0.075	0.514
Tea flavonoids	0.265	0.070	0.654
Kaempferol	0.273	0.070	0.654
Delphinidin	0.314	0.069	0.685
Total flavonoids	0.326	0.069	0.685
Apigenin	0.343	0.069	0.685
Epicatechin	0.376	0.068	0.712
% Flavones (excluding tea)	0.411	0.068	0.740
% Flavan-3-ols (excluding tea)	0.434	0.067	0.744
Total flavonoids (excluding tea)	0.554	0.065	0.905
Cyanidin	0.579	0.064	0.905
Catechin	0.679	0.063	0.967
Ouercetin	0.696	0.063	0.967
Pelargonidin	0.702	0.062	0.967
Naringenin	0.726	0.063	0.967
Isorhamnetin	0.787	0.060	0.977
Myricetin	0.791	0.061	0.977
Luteolin	0.815	0.061	0.977
Epicatechin-3-gallate	0.907	0.058	0.994
Epigallocatecin	0.913	0.058	0.994
Hesperetin	0.920	0.058	0.994
% Flavonols (excluding tea)	0.944	0.058	0.994
% Flavanones (excluding tea)	0.986	0.055	0.994
Epigallocatechin-3-gallate	0.994	0.053	0.994

^1^ Flavonoid-specific food frequency questionnaire validated in the Australian population; ^2^ Adonis (R package vegan), bolded *p* values < 0.05; ^3^ mt.rawp2adjp using “BH” (“fdr”) (R package multtest), bolded *q* values < 0.3.

**Table 2:** Associations between flavonoid intakes ^1^ and gut microbiome based on weighted UniFrac distances.

Variable	*p* Value ^2^	*R*^2^ ^2^	*q* Value ^3^
% Flavones (excluding tea)	0.013	0.184	0.227
% Flavonols (excluding tea)	0.016	0.151	0.227
Apigenin	0.028	0.163	0.227
Kaempferol	0.029	0.145	0.227
Gallocatechin	0.053	0.131	0.227
Theaflavin-3′-gallate	0.053	0.130	0.227
Theaflavin-3-gallate	0.056	0.130	0.227
Thearubigins	0.057	0.130	0.227
Theaflavin digallate	0.059	0.130	0.227
Theaflavin	0.063	0.130	0.227
Tea flavonoids	0.087	0.113	0.285
Total flavonoids	0.098	0.110	0.291
Cocoa flavonoids	0.107	0.107	0.291
Hesperetin	0.113	0.105	0.291
Eriodictyol	0.127	0.103	0.305
Pelargonidin	0.149	0.103	0.318
Cyanidin	0.150	0.099	0.318
Myricetin	0.165	0.096	0.324
% Anthocyanidins (excluding tea)	0.171	0.095	0.324
Delphinidin	0.224	0.085	0.402
% Flavanones (excluding tea)	0.313	0.075	0.537
Epigallocatecin	0.341	0.071	0.542
Epicatechin-3-gallate	0.346	0.072	0.542
Catechin	0.445	0.065	0.652
% Flavan-3-ols (excluding tea)	0.453	0.064	0.652
Epicatechin	0.473	0.063	0.654
Peonidin	0.495	0.060	0.659
Petunidin	0.533	0.055	0.667
Total flavonoids (excluding tea)	0.544	0.059	0.667
Malvidin	0.559	0.053	0.667
Ouercetin	0.574	0.054	0.667
Wine flavonoids	0.617	0.048	0.692
Naringenin	0.634	0.050	0.692
Epigallocatechin-3-gallate	0.704	0.044	0.745
Isorhamnetin	0.811	0.036	0.834
Luteolin	0.852	0.037	0.852

^1^ Flavonoid-specific food frequency questionnaire validated in the Australian population; ^2^ Adonis (R package vegan), bolded *p* values < 0.05; ^3^ mt.rawp2adjp using “BH” (“fdr”) (R package multtest), bolded *q* values < 0.3

## Supplementary Materials

The following are available online at http://www.mdpi.com/2072-6643/10/9/1264/s1. Table S1: Subject clinical characteristics, Table S2: Faecal sample DNA sequencing primers and conditions, Table S3: Flavonoid intakes of subjects over the previous year, Table S4: Taxa composing ≥75% of the gut microbiome at different taxonomic levels; Figure S1: The genus level gut microbiota composition and chao1 and Shannon indices in a group of adults with CF, Figure S2: Heatmap showing correlations between gallocatechin intakes and gut bacterial taxa, Figure S3: Major food sources of tea flavonoids in a group of free-living adults with CF; Supplementary notes: a flavonoid-specific food frequency questionnaire.
