# Supplementary material for: Associations between Flavonoid Intakes and Gut Microbiota in a Group of Adults with Cystic Fibrosis
Source: Nutrients. 2018 Sep 7;10(9):1264. doi: 10.3390/nu10091264 (PMC6164979; doi:10.3390/nu10091264)
Supplement: Supplementary file 1 [file nutrients-10-01264-s001.pdf]

SUPPLEMENTARY TABLES

Table S1 Subject clinical characteristics

Demographic/clinical variable											
Continuous variable											
		Men (n=10)					Women (n=6)				
	unit	mean	sd	median	min	max	mean	sd	median	min	max
Age	years	32.2	9.1	30.5	23.0	52.0	37.3	11.7	34.0	26.0	59.0
BMI	kg/m^2	23.2	3.9	23.7	17.1	30.1	23.9	3.5	24.6	19.0	28.4
FEV1	%	66.8	32.5	51.0	25.0	120.0	73.0	31.5	77.0	30.0	118.0
Categorical variable											
		No. of persons					No. of persons				
Location(Sydney)		4					4				
Pancreatic insufficiency		9					5				
P.aeruginosa colonisation		9					3				
CFRD		2					2				

Table S2 Faecal sample DNA sequencing primers and conditions^

Amplicon Name	Forward Sequence	Reverse sequence	PCR conditions
16S 27F-519R	AdptB_27F_U - AGAGTTTGATCMTGGCTCAG	AdptA_519R_1 - GWATTACCGCGGCKGCTG	94*C for 3min; 34x (94*C for 45s, 50*C for 60s, 72*C for 60s); 72*C for 7 min.

^ Information provided by the Australian Genomic Research Facility

Table S3 Flavonoid intakes of subjects over the previous year*

	Men (n=10)					Women (n=6)				
Flavonoid (mg/day)	mean	sd	median	min	max	mean	sd	median	min	max
Quercetin	29.2	18.8	28.5	5.7	77.3	29.6	19.8	25.9	8.7	65.0
Isorhamnetin	0.3	0.7	0.0	0.0	2.2	0.2	0.4	0.1	0.0	1.2
Kaempferol	2.5	2.5	1.3	0.3	6.7	6.2	4.8	6.4	0.6	13.4
Myricetin	0.9	0.8	0.6	0.1	2.2	2.7	1.9	2.8	0.2	5.1
Apigenin	0.4	0.6	0.2	0.0	2.2	0.9	1.3	0.5	0.0	3.6
Luteolin	0.2	0.2	0.2	0.1	0.6	0.4	0.2	0.3	0.1	0.6
Catechin	9.8	5.8	10.1	1.5	18.3	11.4	6.3	12.0	1.9	19.2
Gallocatechin	1.3	2.0	0.3	0.0	5.2	3.8	4.6	2.1	0.0	12.4
Epicatechin	22.2	12.9	18.1	5.6	49.1	23.6	9.8	22.0	14.3	39.9
Epigallocatechin	9.8	13.6	3.4	0.0	35.9	46.9	40.2	42.2	0.6	105.3
Epicatechin-3-gallate	6.6	10.0	1.1	0.0	26.0	34.1	29.6	30.5	0.0	77.3
Epigallocatechin-3-gallate	11.9	16.9	1.8	0.0	45.7	92.5	102.5	65.7	0.1	280.2
Theaflavin	1.6	2.6	0.3	0.0	6.5	4.8	5.8	2.8	0.0	15.6
Theaflavin-3,3'-digallate	1.8	2.9	0.3	0.0	7.2	5.3	6.4	2.9	0.0	17.3
Theaflavin-3'-gallate	1.6	2.5	0.3	0.0	6.2	4.5	5.5	2.5	0.0	14.9
Theaflavin-3-gallate	1.3	2.1	0.2	0.0	5.2	3.8	4.6	2.1	0.0	12.4
Thearubigins	84.1	134.0	14.1	0.0	335.5	245.4	296.6	137.3	0.0	804.9
Naringenin	6.3	6.8	3.9	0.1	22.2	6.8	10.3	2.9	0.0	27.3
Hesperetin	12.4	12.4	8.3	0.1	38.6	10.4	11.8	5.5	0.0	30.3
Eriodictyolfruits	0.5	0.9	0.2	0.0	3.1	0.3	0.2	0.3	0.0	0.5
Cyanidin	9.6	11.7	5.3	0.4	40.5	4.6	3.3	4.3	0.4	8.3
Delphinidin	2.1	1.6	2.2	0.0	4.8	2.2	2.4	1.8	0.0	5.9
Pelargonidin	0.0	0.0	0.0	0.0	0.1	0.0	0.0	0.0	0.0	0.0
Peonidin	0.9	1.1	0.8	0.0	3.5	0.8	0.7	0.7	0.0	1.7
Malvidin	4.8	8.2	2.9	0.0	26.6	4.4	4.0	3.8	0.0	10.8
Petunidin	0.9	1.4	0.6	0.0	4.5	0.8	0.7	0.6	0.0	1.9
Total flavonoids	223.3	174.2	155.1	45.8	535.1	546.3	406.5	555.0	49.4	1198.0
Tea flavonoids	127.3	198.9	20.6	0.0	505.3	478.5	426.4	443.3	0.0	1181.2

Wine flavonoids	8.1	22.4	0.0	0.0	71.3	7.1	11.0	4.5	0.0	29.0
Cocoa flavonoids	4.9	13.5	0.6	0.0	43.2	0.0	0.1	0.0	0.0	0.2
Total flavonoids (excluding tea)	95.9	64.0	87.7	29.8	246.7	67.8	41.6	68.5	16.8	113.9
^Flavonols% (excluding tea)	30.7	8.1	32.2	17.3	39.8	35.8	17.8	34.7	8.6	56.6
^Flavones% (excluding tea)	0.9	0.7	0.6	0.1	2.1	1.2	1.3	0.7	0.2	3.6
^Flavan-3-ols% (excluding tea)	30.4	11.5	24.9	20.5	50.3	26.8	12.9	27.3	10.2	43.2
^Flavanones% (excluding tea)	19.1	17.6	13.9	1.1	60.2	20.0	17.3	13.5	0.3	41.9
^Anthocyanidins% (excluding tea)	19.0	11.8	19.0	1.3	36.6	16.3	7.9	17.1	2.5	26.9

*estimated using a flavonoid-specific food frequency questionnaire validated in a young Australian population [32]

^Contribution of these flavonoid subclasses to total flavonoid intakes excluding contribution of tea

Table S4 Taxa composing ≥75% of the gut micriobiome at different taxonomic levels

Taxa	Mean % Gut microbiome at each level
Genus	
p_Firmicutes;c_Clostridia;o_Clostridiales;f_Lachnospiraceae;g_Blautia	16.5%
p_Firmicutes;c_Clostridia;o_Clostridiales;f_Lachnospiraceae;g_Coprococcus	11.0%
p_Firmicutes;c_Clostridia;o_Clostridiales;f_Lachnospiraceae;Other	9.1%
p_Firmicutes;c_Clostridia;o_Clostridiales;f_Lachnospiraceae;g_	7.4%
p_Bacteroidetes;c_Bacteroidia;o_Bacteroidales;f_Bacteroidaceae;g_Bacteroides	4.8%
p_Firmicutes;c_Bacilli;o_Lactobacillales;f_Streptococcaceae;g_Streptococcus	4.5%
p_Firmicutes;c_Clostridia;o_Clostridiales;f_Clostridiaceae;g_	4.3%
p_Firmicutes;c_Bacilli;o_Lactobacillales;f_Lactobacillaceae;g_Lactobacillus	4.3%
p_Firmicutes;c_Clostridia;o_Clostridiales;f_Ruminococcaceae;g_	4.2%
p_Firmicutes;c_Clostridia;o_Clostridiales;Other;Other	3.8%
p_Firmicutes;c_Clostridia;o_Clostridiales;f_;g_	2.8%
p_Firmicutes;c_Clostridia;o_Clostridiales;f_Lachnospiraceae;g_Dorea	2.7%
Family	
p_Firmicutes;c_Clostridia;o_Clostridiales;f_Lachnospiraceae	48.6%
p_Firmicutes;c_Clostridia;o_Clostridiales;f_Clostridiaceae	6.9%
p_Firmicutes;c_Clostridia;o_Clostridiales;f_Ruminococcaceae	6.1%
p_Bacteroidetes;c_Bacteroidia;o_Bacteroidales;f_Bacteroidaceae	4.8%
p_Actinobacteria;c_Coriobacteriia;o_Coriobacteriales;f_Coriobacteriaceae	4.8%
p_Firmicutes;c_Bacilli;o_Lactobacillales;f_Streptococcaceae	4.5%
Order	
p_Firmicutes;c_Clostridia;o_Clostridiales	72.0%
p_Firmicutes;c_Bacilli;o_Lactobacillales	10.2%
Class	
p_Firmicutes;c_Clostridia	72.0%
p_Firmicutes;c_Bacilli	10.9%
Phylum	
p_Firmicutes	86.4%

SUPPLEMENTARY FIGURES

a

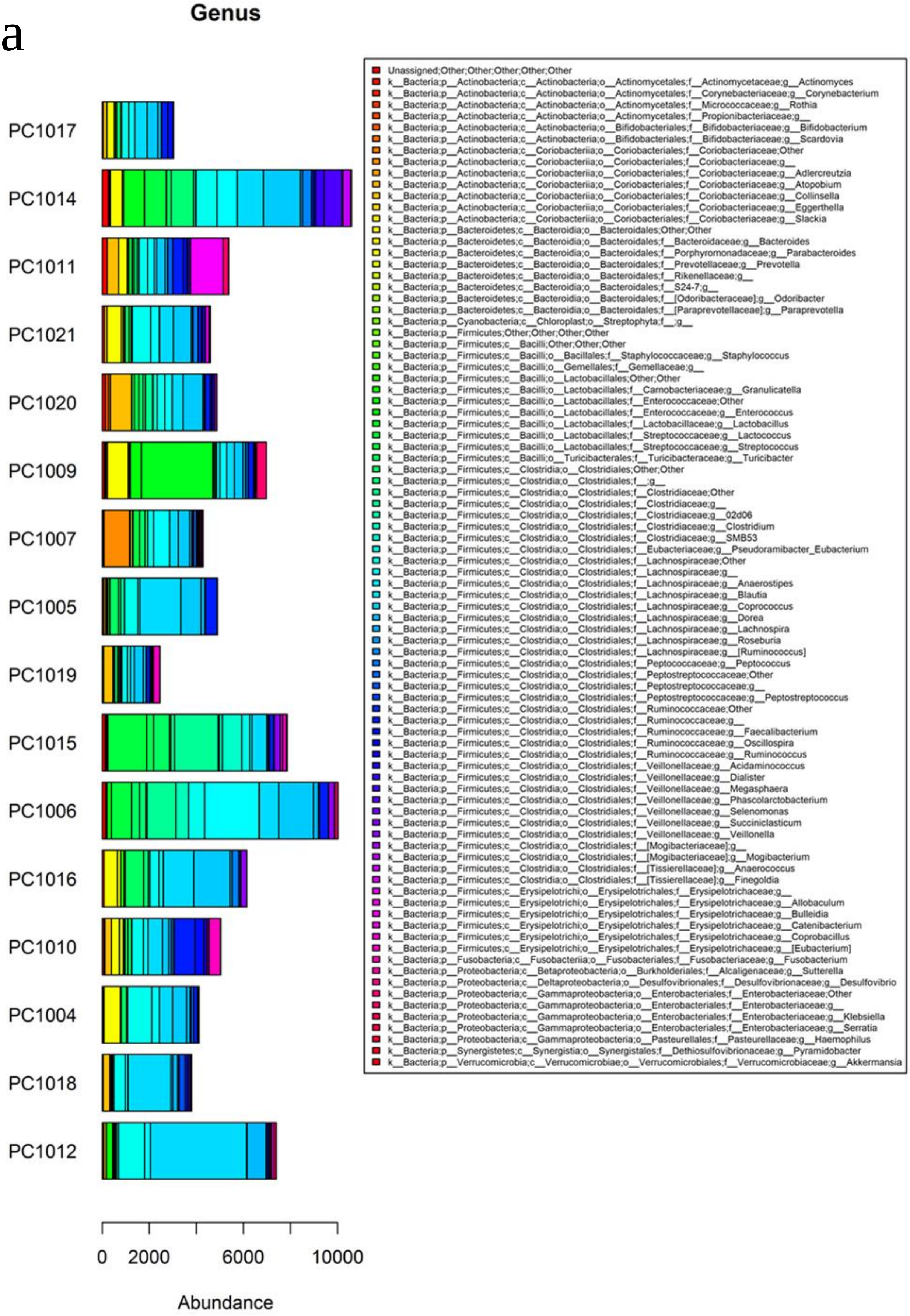

b

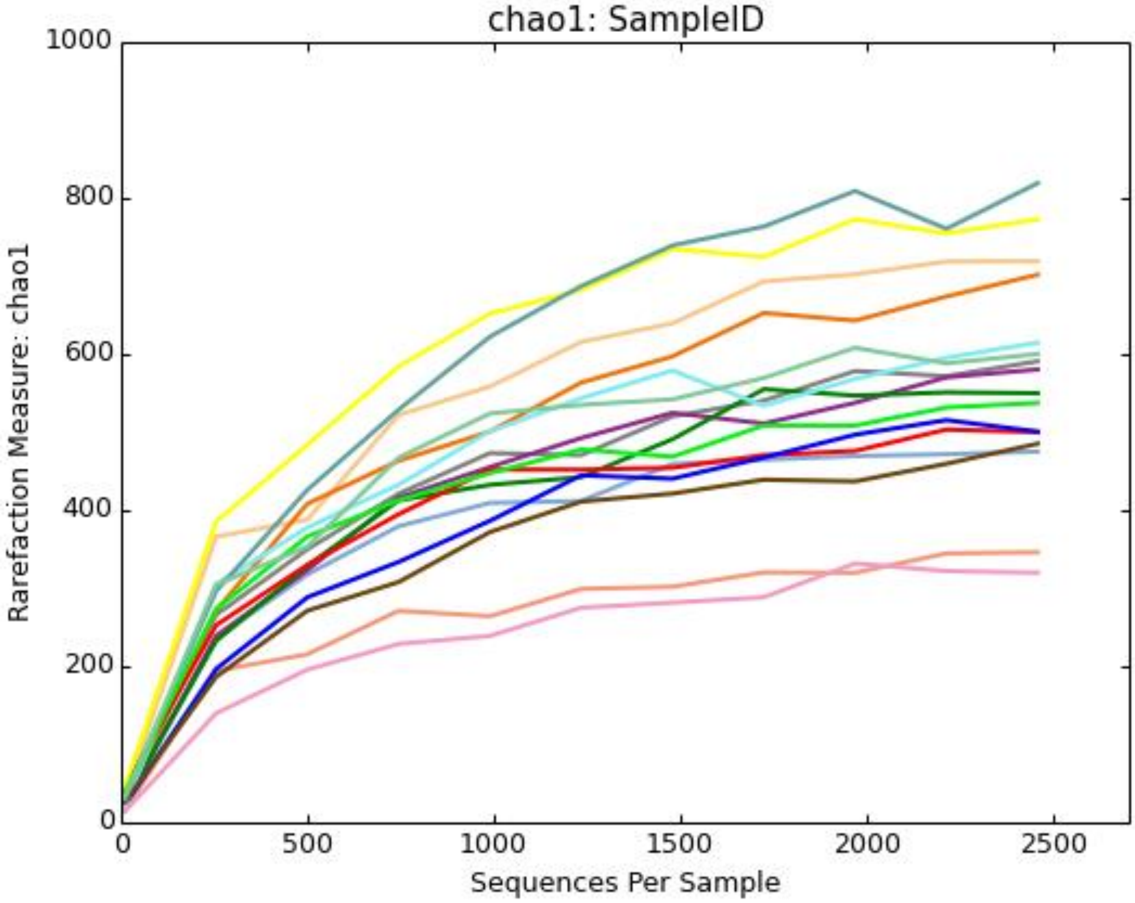
chao1: SampleID
Rarefaction Measure: chao1
Sequences Per Sample
0
200
400
600
800
1000
0
500
1000
1500
2000
2500

C

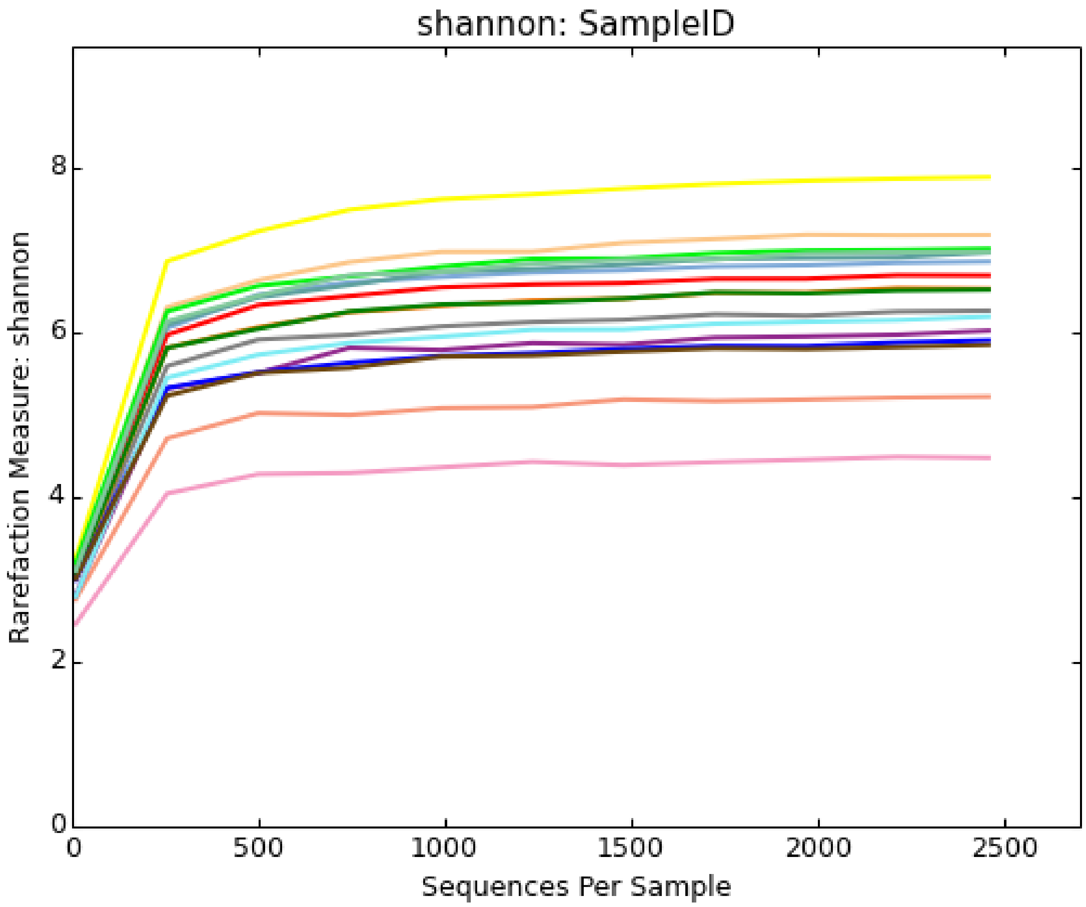

Figure S1 The genus level gut microbiota composition (a), and chao1 index (b) and Shannon index (c) (rarefied at a depth of 2466) in a group of adults with CF

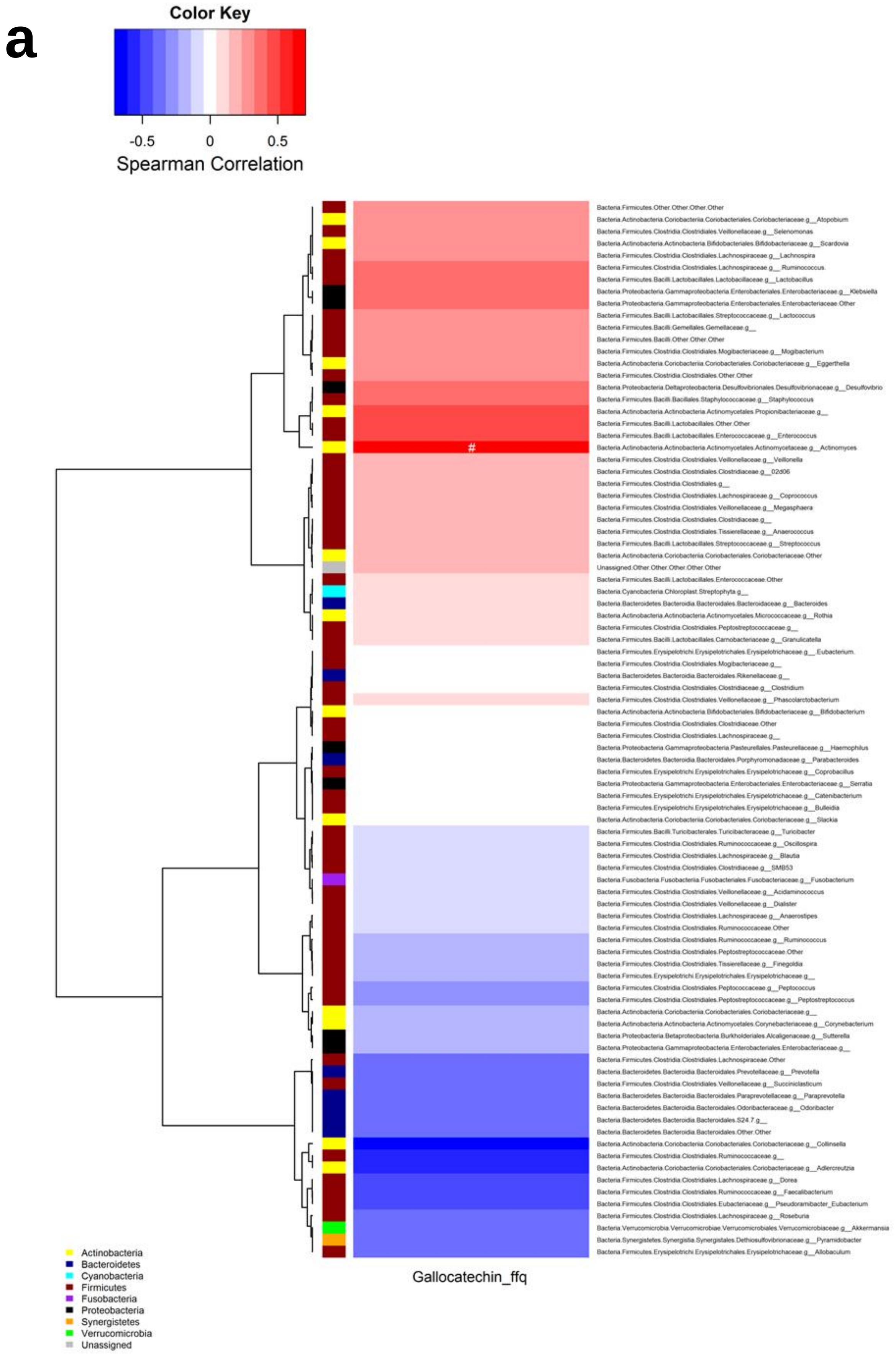

a
Color Key
-0.5
0
0.5
Spearman Correlation
Bacteria.Firmicutes.Other.Other.Other.Other
Bacteria.Actinobacteria.Coriobacteriia.Coriobacteriales.Coriobacteriaceae.g__Atopobium
Bacteria.Firmicutes.Clostridia.Clostridiales.Veillonellaceae.g__Selenomonas
Bacteria.Actinobacteria.Actinobacteria.Bifidobacteriales.Bifidobacteriaceae.g__Scardovia
Bacteria.Firmicutes.Clostridia.Clostridiales.Lachnospiraceae.g__Lachnospira
Bacteria.Firmicutes.Clostridia.Clostridiales.Lachnospiraceae.g__Ruminococcus.
Bacteria.Firmicutes.Bacilli.Lactobacillales.Lactobacillaceae.g__Lactobacillus
Bacteria.Proteobacteria.Gammaproteobacteria.Enterobacteriales.Enterobacteriaceae.g__Klebsiella
Bacteria.Proteobacteria.Gammaproteobacteria.Enterobacteriales.Enterobacteriaceae.Other
Bacteria.Firmicutes.Bacilli.Lactobacillales.Streptococcaceae.g__Lactococcus
Bacteria.Firmicutes.Bacilli.Gemellales.Gemellaceae.g__
Bacteria.Firmicutes.Bacilli.Other.Other.Other
Bacteria.Firmicutes.Clostridia.Clostridiales.Mogibacteriaceae.g__Mogibacterium
Bacteria.Actinobacteria.Coriobacteriia.Coriobacteriales.Coriobacteriaceae.g__Eggerthella
Bacteria.Firmicutes.Clostridia.Clostridiales.Other.Other
Bacteria.Proteobacteria.Deltaproteobacteria.Desulfovibrionales.Desulfovibrionaceae.g__Desulfovibrio
Bacteria.Firmicutes.Bacilli.Bacillales.Staphylococcaceae.g__Staphylococcus
Bacteria.Actinobacteria.Actinobacteria.Actinomycetales.Propionibacteriaceae.g__
Bacteria.Firmicutes.Bacilli.Lactobacillales.Other.Other
Bacteria.Firmicutes.Bacilli.Lactobacillales.Enterococcaceae.g__Enterococcus
#
Bacteria.Actinobacteria.Actinobacteria.Actinomycetales.Actinomycetaceae.g__Actinomyces
Bacteria.Firmicutes.Clostridia.Clostridiales.Veillonellaceae.g__Veillonella
Bacteria.Firmicutes.Clostridia.Clostridiales.Clostridiaceae.g__02d06
Bacteria.Firmicutes.Clostridia.Clostridiales.g__
Bacteria.Firmicutes.Clostridia.Clostridiales.Lachnospiraceae.g__Coprococcus
Bacteria.Firmicutes.Clostridia.Clostridiales.Veillonellaceae.g__Megasphaera
Bacteria.Firmicutes.Clostridia.Clostridiales.Clostridiaceae.g__
Bacteria.Firmicutes.Clostridia.Clostridiales.Tissierellaceae.g__Anaerococcus
Bacteria.Firmicutes.Bacilli.Lactobacillales.Streptococcaceae.g__Streptococcus
Bacteria.Actinobacteria.Coriobacteriia.Coriobacteriales.Coriobacteriaceae.Other
Unassigned.Other.Other.Other.Other.Other
Bacteria.Firmicutes.Bacilli.Lactobacillales.Enterococcaceae.Other
Bacteria.Cyanobacteria.Chloroplast.Streptophyta.g__
Bacteria.Bacteroidetes.Bacteroidia.Bacteroidales.Bacteroidaceae.g__Bacteroides
Bacteria.Actinobacteria.Actinobacteria.Actinomycetales.Micrococcaceae.g__Rothia
Bacteria.Firmicutes.Clostridia.Clostridiales.Peptostreptococcaceae.g__
Bacteria.Firmicutes.Bacilli.Lactobacillales.Carnobacteriaceae.g__Granulicatella
Bacteria.Firmicutes.Erysipelotrichi.Erysipelotrichales.Erysipelotrichaceae.g__Eubacterium.
Bacteria.Firmicutes.Clostridia.Clostridiales.Mogibacteriaceae.g__
Bacteria.Bacteroidetes.Bacteroidia.Bacteroidales.Rikenellaceae.g__
Bacteria.Firmicutes.Clostridia.Clostridiales.Clostridiaceae.g__Clostridium
Bacteria.Firmicutes.Clostridia.Clostridiales.Veillonellaceae.g__Phascolarctobacterium
Bacteria.Actinobacteria.Actinobacteria.Bifidobacteriales.Bifidobacteriaceae.g__Bifidobacterium
Bacteria.Firmicutes.Clostridia.Clostridiales.Clostridiaceae.Other
Bacteria.Firmicutes.Clostridia.Clostridiales.Lachnospiraceae.g__
Bacteria.Proteobacteria.Gammaproteobacteria.Pasteurellales.Pasteurellaceae.g__Haemophilus
Bacteria.Bacteroidetes.Bacteroidia.Bacteroidales.Porphyromonadaceae.g__Parabacteroides
Bacteria.Firmicutes.Erysipelotrichi.Erysipelotrichales.Erysipelotrichaceae.g__Coprobacillus
Bacteria.Proteobacteria.Gammaproteobacteria.Enterobacteriales.Enterobacteriaceae.g__Serratia
Bacteria.Firmicutes.Erysipelotrichi.Erysipelotrichales.Erysipelotrichaceae.g__Catenibacterium
Bacteria.Firmicutes.Erysipelotrichi.Erysipelotrichales.Erysipelotrichaceae.g__Bulleidia
Bacteria.Actinobacteria.Coriobacteriia.Coriobacteriales.Coriobacteriaceae.g__Slackia
Bacteria.Firmicutes.Bacilli.Turicibacterales.Turicibacteraceae.g__Turicibacter
Bacteria.Firmicutes.Clostridia.Clostridiales.Ruminococcaceae.g__Oscillospira
Bacteria.Firmicutes.Clostridia.Clostridiales.Lachnospiraceae.g__Blautia
Bacteria.Firmicutes.Clostridia.Clostridiales.Clostridiaceae.g__SMB53
Bacteria.Fusobacteria.Fusobacteriia.Fusobacteriales.Fusobacteriaceae.g__Fusobacterium
Bacteria.Firmicutes.Clostridia.Clostridiales.Veillonellaceae.g__Acidaminococcus
Bacteria.Firmicutes.Clostridia.Clostridiales.Veillonellaceae.g__Dialister
Bacteria.Firmicutes.Clostridia.Clostridiales.Lachnospiraceae.g__Anaerostipes
Bacteria.Firmicutes.Clostridia.Clostridiales.Ruminococcaceae.Other
Bacteria.Firmicutes.Clostridia.Clostridiales.Ruminococcaceae.g__Ruminococcus
Bacteria.Firmicutes.Clostridia.Clostridiales.Peptostreptococcaceae.Other
Bacteria.Firmicutes.Clostridia.Clostridiales.Tissierellaceae.g__Finegoldia
Bacteria.Firmicutes.Erysipelotrichi.Erysipelotrichales.Erysipelotrichaceae.g__
Bacteria.Firmicutes.Clostridia.Clostridiales.Peptococcaceae.g__Peptococcus
Bacteria.Firmicutes.Clostridia.Clostridiales.Peptostreptococcaceae.g__Peptostreptococcus
Bacteria.Actinobacteria.Coriobacteriia.Coriobacteriales.Coriobacteriaceae.g__
Bacteria.Actinobacteria.Actinobacteria.Actinomycetales.Corynebacteriaceae.g__Corynebacterium
Bacteria.Proteobacteria.Betaproteobacteria.Burkholderiales.Alcaligenaceae.g__Sutterella
Bacteria.Proteobacteria.Gammaproteobacteria.Enterobacteriales.Enterobacteriaceae.g__
Bacteria.Firmicutes.Clostridia.Clostridiales.Lachnospiraceae.Other
Bacteria.Bacteroidetes.Bacteroidia.Bacteroidales.Prevotellaceae.g__Prevotella
Bacteria.Firmicutes.Clostridia.Clostridiales.Veillonellaceae.g__Succiniclasticum
Bacteria.Bacteroidetes.Bacteroidia.Bacteroidales.Paraprevotellaceae.g__Paraprevotella
Bacteria.Bacteroidetes.Bacteroidia.Bacteroidales.Odoribacteraceae.g__Odoribacter
Bacteria.Bacteroidetes.Bacteroidia.Bacteroidales.S24.7.g__
Bacteria.Bacteroidetes.Bacteroidia.Bacteroidales.Other.Other
Bacteria.Actinobacteria.Coriobacteriia.Coriobacteriales.Coriobacteriaceae.g__Collinsella
Bacteria.Firmicutes.Clostridia.Clostridiales.Ruminococcaceae.g__
Bacteria.Actinobacteria.Coriobacteriia.Coriobacteriales.Coriobacteriaceae.g__Adlercreutzia
Bacteria.Firmicutes.Clostridia.Clostridiales.Lachnospiraceae.g__Dorea
Bacteria.Firmicutes.Clostridia.Clostridiales.Ruminococcaceae.g__Faecalibacterium
Bacteria.Firmicutes.Clostridia.Clostridiales.Eubacteriaceae.g__Pseudoramibacter_Eubacterium
Bacteria.Firmicutes.Clostridia.Clostridiales.Lachnospiraceae.g__Roseburia
Bacteria.Verrucomicrobia.Verrucomicrobiae.Verrucomicrobiales.Verrucomicrobiaceae.g__Akkermansia
Bacteria.Synergistetes.Synergistia.Synergistales.Dethiosulfovibrionaceae.g__Pyramidobacter
Bacteria.Firmicutes.Erysipelotrichi.Erysipelotrichales.Erysipelotrichaceae.g__Allobaculum
Gallocatechin_ffq
Actinobacteria
Bacteroidetes
Cyanobacteria
Firmicutes
Fusobacteria
Proteobacteria
Synergistetes
Verrucomicrobia
Unassigned

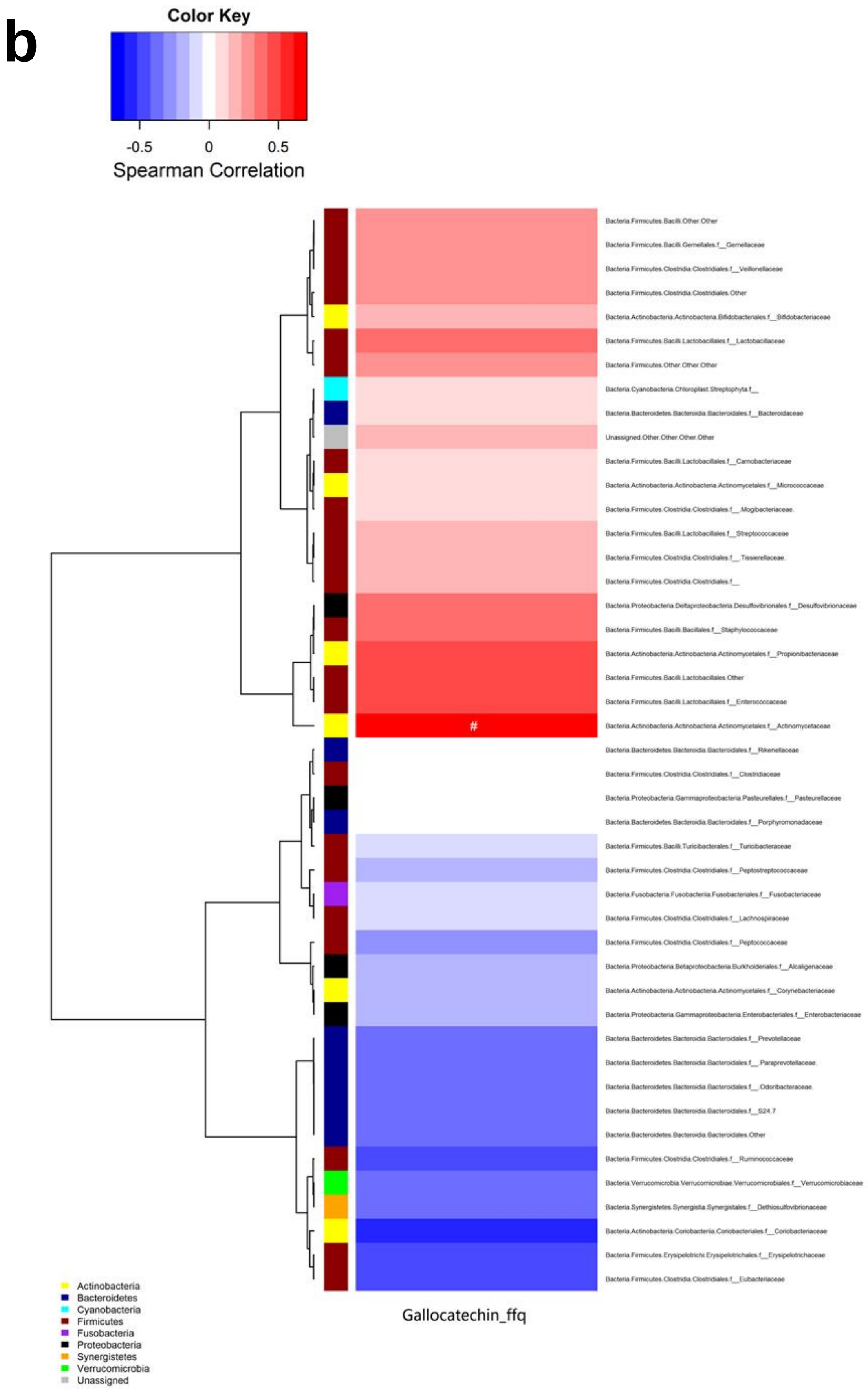
b
Color Key
-0.5
0
0.5
Spearman Correlation
Bacteria.Firmicutes.Bacilli.Other.Other
Bacteria.Firmicutes.Bacilli.Gemellales.f__Gemellaceae
Bacteria.Firmicutes.Clostridia.Clostridiales.f__Veillonellaceae
Bacteria.Firmicutes.Clostridia.Clostridiales.Other
Bacteria.Actinobacteria.Actinobacteria.Bifidobacteriales.f__Bifidobacteriaceae
Bacteria.Firmicutes.Bacilli.Lactobacillales.f__Lactobacillaceae
Bacteria.Firmicutes.Other.Other.Other
Bacteria.Cyanobacteria.Chloroplast.Streptophyta.f__
Bacteria.Bacteroidetes.Bacteroidia.Bacteroidales.f__Bacteroidaceae
Unassigned.Other.Other.Other.Other
Bacteria.Firmicutes.Bacilli.Lactobacillales.f__Carnobacteriaceae
Bacteria.Actinobacteria.Actinobacteria.Actinomycetales.f__Micrococcaceae
Bacteria.Firmicutes.Clostridia.Clostridiales.f__.Mogibacteriaceae.
Bacteria.Firmicutes.Bacilli.Lactobacillales.f__Streptococcaceae
Bacteria.Firmicutes.Clostridia.Clostridiales.f__.Tissierellaceae.
Bacteria.Firmicutes.Clostridia.Clostridiales.f__
Bacteria.Proteobacteria.Deltaproteobacteria.Desulfovibrionales.f__Desulfovibrionaceae
Bacteria.Firmicutes.Bacilli.Bacillales.f__Staphylococcaceae
Bacteria.Actinobacteria.Actinobacteria.Actinomycetales.f__Propionibacteriaceae
Bacteria.Firmicutes.Bacilli.Lactobacillales.Other
Bacteria.Firmicutes.Bacilli.Lactobacillales.f__Enterococcaceae
#
Bacteria.Actinobacteria.Actinobacteria.Actinomycetales.f__Actinomycetaceae
Bacteria.Bacteroidetes.Bacteroidia.Bacteroidales.f__Rikenellaceae
Bacteria.Firmicutes.Clostridia.Clostridiales.f__Clostridiaceae
Bacteria.Proteobacteria.Gammaproteobacteria.Pasteurellales.f__Pasteurellaceae
Bacteria.Bacteroidetes.Bacteroidia.Bacteroidales.f__Porphyromonadaceae
Bacteria.Firmicutes.Bacilli.Turicibacterales.f__Turicibacteraceae
Bacteria.Firmicutes.Clostridia.Clostridiales.f__Peptostreptococcaceae
Bacteria.Fusobacteria.Fusobacteriia.Fusobacteriales.f__Fusobacteriaceae
Bacteria.Firmicutes.Clostridia.Clostridiales.f__Lachnospiraceae
Bacteria.Firmicutes.Clostridia.Clostridiales.f__Peptococcaceae
Bacteria.Proteobacteria.Betaproteobacteria.Burkholderiales.f__Alcaligenaceae
Bacteria.Actinobacteria.Actinobacteria.Actinomycetales.f__Corynebacteriaceae
Bacteria.Proteobacteria.Gammaproteobacteria.Enterobacteriales.f__Enterobacteriaceae
Bacteria.Bacteroidetes.Bacteroidia.Bacteroidales.f__Prevotellaceae
Bacteria.Bacteroidetes.Bacteroidia.Bacteroidales.f__.Paraprevotellaceae.
Bacteria.Bacteroidetes.Bacteroidia.Bacteroidales.f__.Odoribacteraceae.
Bacteria.Bacteroidetes.Bacteroidia.Bacteroidales.f__S24.7
Bacteria.Bacteroidetes.Bacteroidia.Bacteroidales.Other
Bacteria.Firmicutes.Clostridia.Clostridiales.f__Ruminococcaceae
Bacteria.Verrucomicrobia.Verrucomicrobiae.Verrucomicrobiales.f__Verrucomicrobiaceae
Bacteria.Synergistetes.Synergistia.Synergistales.f__Dethiosulfovibrionaceae
Bacteria.Actinobacteria.Coriobacteriia.Coriobacteriales.f__Coriobacteriaceae
Bacteria.Firmicutes.Erysipelotrichi.Erysipelotrichales.f__Erysipelotrichaceae
Bacteria.Firmicutes.Clostridia.Clostridiales.f__Eubacteriaceae
Actinobacteria
Bacteroidetes
Cyanobacteria
Firmicutes
Fusobacteria
Proteobacteria
Synergistetes
Verrucomicrobia
Unassigned
Gallocatechin_ffq

c

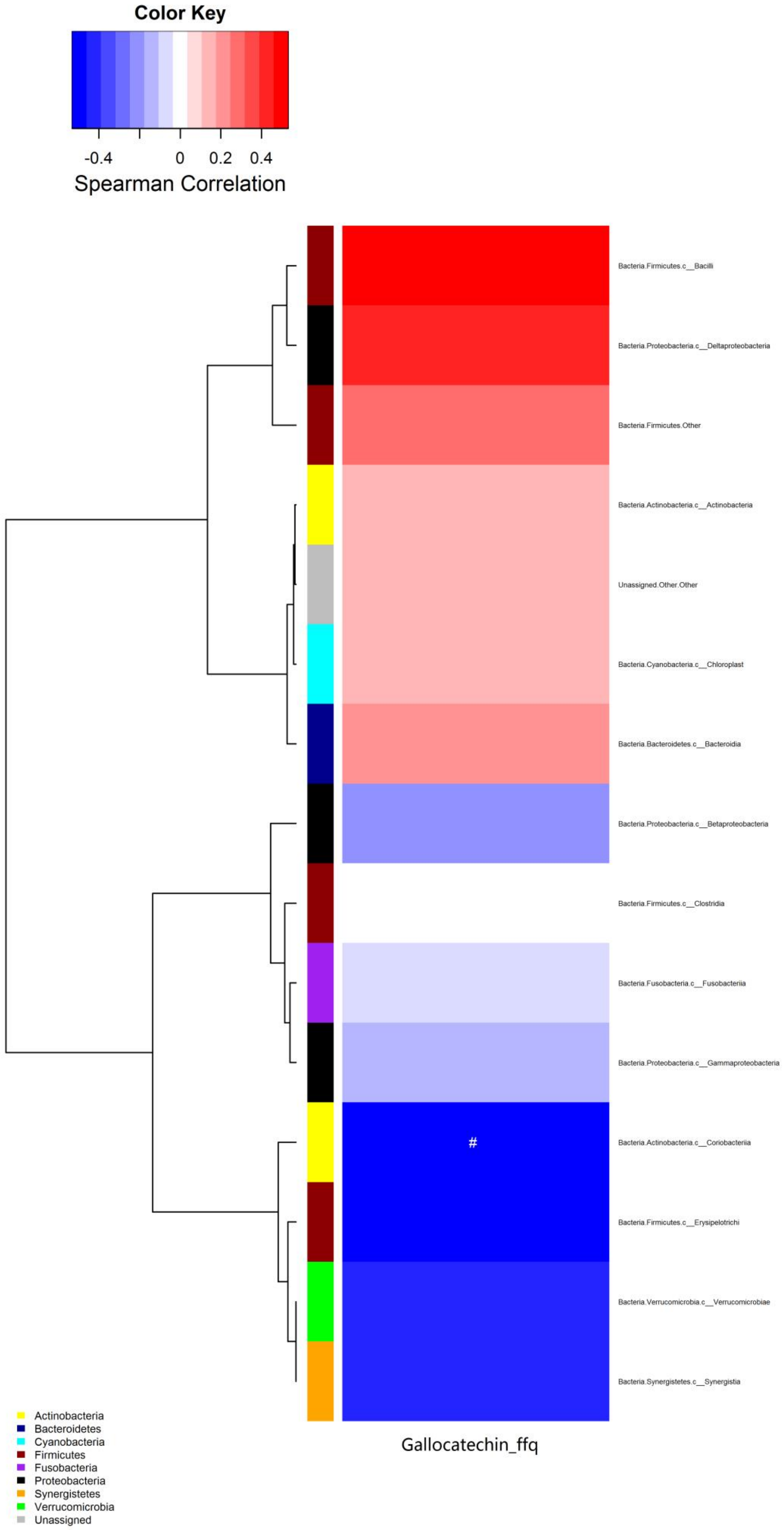

Color Key
-0.4
0
0.2
0.4
Spearman Correlation
Bacteria.Firmicutes.c__Bacilli
Bacteria.Proteobacteria.c__Deltaproteobacteria
Bacteria.Firmicutes.Other
Bacteria.Actinobacteria.c__Actinobacteria
Unassigned.Other.Other
Bacteria.Cyanobacteria.c__Chloroplast
Bacteria.Bacteroidetes.c__Bacteroidia
Bacteria.Proteobacteria.c__Betaproteobacteria
Bacteria.Firmicutes.c__Clostridia
Bacteria.Fusobacteria.c__Fusobacteriia
Bacteria.Proteobacteria.c__Gammaproteobacteria
#
Bacteria.Actinobacteria.c__Coriobacteriia
Bacteria.Firmicutes.c__Erysipelotrichi
Bacteria.Verrucomicrobia.c__Verrucomicrobiae
Bacteria.Synergistetes.c__Synergistia
Gallocatechin_ffq
Actinobacteria
Bacteroidetes
Cyanobacteria
Firmicutes
Fusobacteria
Proteobacteria
Synergistetes
Verrucomicrobia
Unassigned

Figure S2 Heatmap showing correlations between gallocatechin intakes and gut bacterial taxa

Note:

1 Spearman correlations based on significant Adonis tests results at FDR<0.3 with unweighted UniFrac distance at genus level: **a**; family level: **b**; class level: **c**

2 Heatmap generated using heatmap.2 (R package gplots); taxa clustered using “ward.D” method, variables clustered using “single” method

3 “#” denotes significant correlations at FDR<0.3 after adjustment for multiple Spearman tests; raw p values and heatmap with no significant results not shown

4 Variables and relative abundance at different taxonomic levels standardised across samples with a mean = 0, standard deviation =1

5 ffq = Flavonoid-specific food frequency questionnaire validated in the Australian population [32]

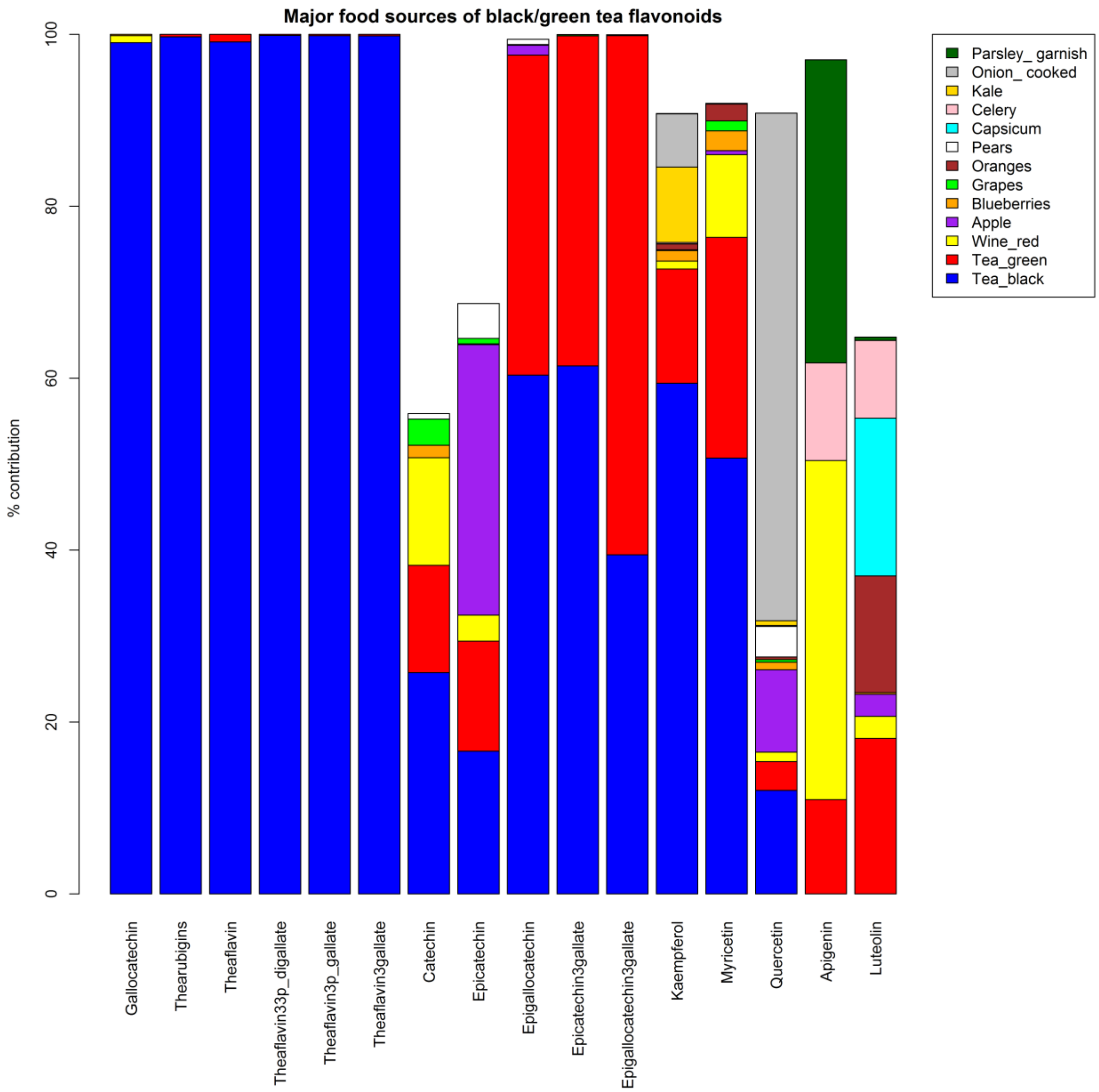

Figure S3 Major food sources of tea flavonoids* in a group of free-living adults with CF

*Estimated by a flavonoid-specific FFQ [32]

SUPPLEMENTARY NOTES

A flavonoid-specific food frequency questionnaire

FFQ

Participant number ………………………   Date …………

Your 1-year dietary patterns

Your **usual** food intake: For each of the foods listed, fill the circle which shows year average* intake over the past year. Pay particular attention to the amount for each food before you answer. (Fill in only **one** circle per row)

* Average over the **whole year**: If you only eat cherries 4 times per week during summer (3 months), then your average intake is once per week.

**Vegetables**	Never, or less than once per month	1-3 per month	1 per week	2-4 per week	1 per day	2-3 per day	4-5 per day	6+ per day
Avocado (½ cup or ½ fruit)	○	○	○	○	○	○	○	○
Bean, broad (½ cup)	○	○	○	○	○	○	○	○
Bean, green (1/2 cup)	○	○	○	○	○	○	○	○
Beetroot (½ cup)	○	○	○	○	○	○	○	○
Broccoli (½ cup)	○	○	○	○	○	○	○	○
Brussels sprout (½ cup)	○	○	○	○	○	○	○	○
Cabbage, green (½ cup)	○	○	○	○	○	○	○	○
Cabbage, red (½ cup)	○	○	○	○	○	○	○	○
Capsicum (½ cup)	○	○	○	○	○	○	○	○
Carrot (½ cup)	○	○	○	○	○	○	○	○
Carrot juice (small glass)	○	○	○	○	○	○	○	○
Cauliflower (½ cup)	○	○	○	○	○	○	○	○
Celery (2-3 sticks)	○	○	○	○	○	○	○	○
Eggplant (Aubergine) (½ cup)	○	○	○	○	○	○	○	○
Endive (1 cup)	○	○	○	○	○	○	○	○
Kale (½ cup)	○	○	○	○	○	○	○	○
Lettuce, common (1 cup)	○	○	○	○	○	○	○	○
Lettuce, iceberg (head) (1 cup)	○	○	○	○	○	○	○	○
Lettuce, cos (leaf) (1 cup)	○	○	○	○	○	○	○	○
Mushroom (½ cup)	○	○	○	○	○	○	○	○
Olive (10 small)	○	○	○	○	○	○	○	○
Onion, as garnish (1 slice)	○	○	○	○	○	○	○	○
Onion, cooked (1 cup)	○	○	○	○	○	○	○	○
Parsley garnish (1 sprig)	○	○	○	○	○	○	○	○
Parsely salad (eg Tabouleh) (½ cup)	○	○	○	○	○	○	○	○
Peas (½ cup)	○	○	○	○	○	○	○	○
Rhubarb (½ cup)	○	○	○	○	○	○	○	○
Silverbeet (1 cup)	○	○	○	○	○	○	○	○
Spinach, Chinese (1 cup)	○	○	○	○	○	○	○	○
Spinach, English (1 cup)	○	○	○	○	○	○	○	○
Sweet potato (½ cup)	○	○	○	○	○	○	○	○
Tomato (2 slices)	○	○	○	○	○	○	○	○
Tomato-based sauce (eg Spaghetti) (½ cup)	○	○	○	○	○	○	○	○
Zucchini (½ cup)	○	○	○	○	○	○	○	○

FFQ

Fruit	Never, or less than once per month	1-3 per month	1 per week	2-4 per week	1 per day	2-3 per day	4-5 per day	6+ per day
Apples (1 whole)	○	○	○	○	○	○	○	○
Apple juice (small glass or 160ml)	○	○	○	○	○	○	○	○
Apricot (1 whole)	○	○	○	○	○	○	○	○
Blueberries (½ cup)	○	○	○	○	○	○	○	○
Cherries (½ cup)	○	○	○	○	○	○	○	○
Grapes (½ cup)	○	○	○	○	○	○	○	○
Grape juice (small glass or 160ml)	○	○	○	○	○	○	○	○
Grapefruit (½ fruit)	○	○	○	○	○	○	○	○
Grapefruit juice (small glass or 160ml)	○	○	○	○	○	○	○	○
Lemon (1 slice)	○	○	○	○	○	○	○	○
Lemon (juice from ½ fruit)	○	○	○	○	○	○	○	○
Mandarin (1 whole)	○	○	○	○	○	○	○	○
Mango (1 whole)	○	○	○	○	○	○	○	○
Nectarine (1 fresh)	○	○	○	○	○	○	○	○
Oranges (1 whole)	○	○	○	○	○	○	○	○
Orange juice (small glass or 160ml)	○	○	○	○	○	○	○	○
Pears (1 whole)	○	○	○	○	○	○	○	○

Beverages	Never, or less than once per month	1-3 per month	1 per week	2-4 per week	1 per day	2-3 per day	4-5 per day	6+ per day
Chocolate (dark) (5 squares or 20g)	○	○	○	○	○	○	○	○
Chocolate (milk) (5 squares or 20g)	○	○	○	○	○	○	○	○
Cocoa (1 tsp)	○	○	○	○	○	○	○	○
Coffee, brewed (small cup or 160ml)	○	○	○	○	○	○	○	○
Coffee, instant (1 tsp)	○	○	○	○	○	○	○	○
Tea, black (small cup or 160ml)	○	○	○	○	○	○	○	○
Tea, green (small cup or 160ml)	○	○	○	○	○	○	○	○
Tomato juice (small glass or 160ml)	○	○	○	○	○	○	○	○
Vegetable juice (eg. V-8) (small glass)	○	○	○	○	○	○	○	○
Wine, red (glass or 160ml)	○	○	○	○	○	○	○	○
Wine, white (glass or 160ml)	○	○	○	○	○	○	○	○

If you have any queries, please feel free to contact:

© Dr Shawn Somerset 2011
